# Comparative heat stress responses of three hot pepper (*Capsicum annuum* L.) genotypes differing temperature sensitivity

**DOI:** 10.1038/s41598-023-41418-5

**Published:** 2023-08-30

**Authors:** Min Kyoung Kim, Hyo Bong Jeong, Nari Yu, Bo Mi Park, Won Byoung Chae, Oak Jin Lee, Hye Eun Lee, Sumin Kim

**Affiliations:** 1 Department of Environmental Horticulture and Landscape Architecture, Environmental Horticulture, Dankook University, Cheonan, 31116 Republic of Korea; 2 Vegetable Research Division, National Institute of Horticultural and Herbal Science, RDA, Wanju, 55365 Republic of Korea

**Keywords:** Plant physiology, Plant stress responses, Heat

## Abstract

As global temperatures have steadily increased over past decades, studying of the impacts of heat stress on morpho-physiological traits and economic yields of horticultural crops have been increasingly gained attentions by many scientists and farmers. Hot pepper (*Capsicum annuum* L.) is an important vegetable crop mostly grown in open-fields in South Korea. In this study, the impacts of prolonged heat stress on three hot pepper genotypes differing by levels of stress susceptibility were evaluated. The study was conducted in two different temperature-controlled greenhouses for 75 days. 48 days old plants were grown in control and heat-treated greenhouses where the temperatures had been set at 30 °C and 35 °C during the day for 75 days, respectively. Morphological, physiological, and nutrient characteristics of three accessions were measured. All hot pepper accessions were enabled to recover from prolonged heat stress exposures within approximately a month. The phenomenon of recovery was observed in some significant morphological and physiological characteristics. For example, the plant growth rate and photosynthesis rate significantly increased after 40th days of heat treatment. The heat stress sensitivity varied between genotypes. The plants that produced more fruits over biomass at early stage of heat treatment had relatively slow recovery, resulting in the largest yield loss. This key morphological characteristic can be used for future breeding program to adapt the prolonged heat stress.

## Introduction

As temperature is one of significant environmental factors that influences plant growth and development processes, including photosynthesis, transpiration, respiration, pollination, and fruit development, steadily rising temperature has been a major abiotic stress limit the growth and development of plants. During the stages of the reproductive organ development (e.g. flowering, pollinating, and fertilization), in particular, plants were mostly sensitive to heat stress, resulting in reduced fruit yields^1,2^. Due to global warming, earth temperature has risen by average of at least 1.1 °C since 1880s^3,4^. In 2021, the average temperature across global surface was 0.84 °C above the twentieth century average^5^. In Korea, according to Greenpeace report^6^, the number of days when air temperatures was above 33 °C has been continuously increased since 1980s. In recent years, the number of 35 °C days was significantly increased^6^. As many meteorologists expected that such warmer events will continuously increase over the next 30 years^7–9^, understanding the potential impacts of heat stress on plant growth and fruit yields will play a key role in development of adaptations strategies to offset these effects.

Hot pepper (*Capsicum annuum* L.) is an economically important fruit-bearing vegetable in Korea due to major spice in Korean cooking. In 2022, the total production of hot pepper in Korea was around 92,757 tons^10^. The majority of hot peppers are produced in the open-field on raised bed using drip irrigation and mulch in Korea. Thus, the weather conditions (e.g. temperature, precipitation, and etc.) of the cultivated area influences directly the fruit yields of the crop. Although peppers have been well adapted to hot climates, the increased number of hot days (above 33 °C) has been a great challenge in achieving sustainable pepper production in Korea. The optimal temperature range of fruit formation and quality is between 21 and 29 °C^11^. When temperature exceed 32 °C, the hot pepper growth can be slow; blossom end rot (BER) can be observed on fruits; and fruit-set ceases may emerge, with lower yields^12^.

The responses of plants toward heat stress highly depend on their degree and duration, and plant’s developmental stage^13^. The heat stress directly or indirectly damages plant’s function, resulting in morphophysiological changes, abnormal growth phases and metabolic processes^14^ and yield reduction^15^. These growth abnormalities may be continuously observed or not shown in plants while plants expose to heat stress, and every plant or genotype have evolved with their own tolerances or mechanisms to manage heat stress conditions^16^. Some tolerant genotypes within several crop species, in contrast, had highest yields under high temperature conditions^17–19^. Several studies have reported genotypic differences in tolerance/susceptibility of hot peppers by comparisons in the rates of germination and seedling growth^20,21^, crop growth, physiology, yields^21^, photosynthesis^20,22^ and pollen tube length and pollen germination and membrane stability^23,24^ between heat-susceptible and heat-tolerant genotypes. According to the these previous studies, increases in duration rates and temperature significantly reduced germination rates, seedling weights, photosynthesis rates, and fruit yields were significantly reduced when temperatures are at least 5 °C above optimal plant growth temperature^25^ or at 42 °C^21^.

Plant responses to heat stress vary by the levels of cumulative of heat’s severity or the amount of heat stress exposures time. Plants can either suffer or protect them in a timely manner. As explained in previous paragraph, when plants subject to heat stress for long period of time, they might experience of heat stress resulting in physiological retardation in a variety of physiological processes, including photosynthesis, respiration, transpiration, membrane thermostability, and osmotic regulation^26^. In contrast, Bäurle^26^ reported that some plants can successfully adapt to the high stressful conditions such as extreme temperatures, drought, salinity, etc. One of strategies for adaptation in stressful condition is priming, where a past stress exposure modifies responses to a later stress event^28–32^. The stress memory takes at different biochemical and morpho-physiological processes, and the stress memory helps plant to respond efficiently to later stress event^27^. Most studies, however, have conducted the experiments monitoring heat stress responses only for relatively short growth periods or only one of growth stage periods (e.g. flowering, seedling, or germination stages). Thus, most studies reported adverse effects of short-term heat stress exposes on the plant growth. There is still limited knowledge on accumulative plant physiological stress induced by extended periods of high temperature, which can be differed from the short-term heat stress responses.

This study aimed to evaluate the association between long-term exposure to high temperature and accumulative plant morphophysiological, photosynthetic, and yield responses in three hot pepper genotypes. Since the susceptibility to heat stress is generally differed by plant genotypes, comparisons in the morphophysiological responses of heat-sensitive and heat-tolerant genotypes to prolonged heat stress will provide insight that can cope with future climate. Changes in nutrient use efficiency under heat stress condition were also studied to understand relationship between heat tolerance and nutrient efficiency.

## Materials and methods

### Plant materials and experimental design

Three hot pepper (*C. annuum* L.) accessions, including PHR5 (accession code of IT235550), PHR18 (accession code of IT286261), and PHR23 (commercial cultivar name of ‘Big Star’), were used in the experiment. PHR5 and PHR23 are known as heat-tolerant accessions, while PHR18 was heat-sensitive accession. The seeds of accessions of PHR5 and PHR23 were obtained from Vegetable Research Division, National Institute of Horticultural and Herbal Science (Wanju, Korea). The origins of PHR5 and PHR23 are Russia (Krasnodar Territory, Kropotkin distr., Ladozh) and Korea (Korean Agricultural Culture Collection, KACC, Wanju, Korea), respectively. Experimental samples of hot peppers, including the collection of plant materials, were collected according to relevant institutions, national and international guidelines and legislation, with the appropriate permission of Agricultural Diversity from the National Academy of Agricultural Sciences (Wanju, Korea). The seeds of PHR23 were obtained from Nongwoo Bio (Suwon, Korea). The seeds of three accessions were sown on March 16th 2022 in plastic trays (54 × 28 cm in size, 6 × 12 cells with pot volume 2.4 L) that were filled with commercial bed soil (‘Bio Sangto’; Seoul, Korea) containing cocopeat (67.5%), peat moss (17%), zeolite (5%), per-lite (10.0%), pH adjuster (0.3%), humectant (0.014%), and fertilizers (0.185%) containing 270 mg kg^−1^ of each of N, P, and K, respectively. The seedlings were grown for 48 days in a glasshouse (26/18 °C in day/night (16/8 h) with relative humidity within 65–70%) at the National Institute of Horticultural and Herbal Science (Wanju, Korea, 35°83′ N, 127°03′ E). On May 3rd, 2022, the hot pepper seedlings were transferred to greenhouses.

The experiment consisted of a split-plot system repeated three times. The main plots were heat treatment, heat stress and control, while the subplots were genotype from three accessions: PHR5, PHR18, and PHR23. The main plots were conducted in two plastic-covered greenhouses. In each greenhouse, subplots were arranged using a randomized complete block design. The subplots consisted of single row plots that were 1.5 m long and contained five transplants spaced 30 cm apart. There were three blocks, and the distance between the single-row plots was 140 cm. The test beds were covered with black plastic mulch film. Prior to the heat treatment, the seedling plants were grown in the greenhouse for two weeks under a ventilation temperature set-point of 30 °C to ensure successful establishment in the new conditions. After two weeks, the air temperature of heat-treated greenhouse was set to 35 °C during the day, while the other greenhouse, serving as the control, remained unchanged. The ventilation system was used to control set-point temperatures in both treated and control greenhouses. The average air temperatures in the control greenhouse remained relatively stable, ranging from 25 to 35 °C. The average temperatures of control and heat-treated greenhouses were 28 °C and 30 °C, respectively. In the heat-treated greenhouse, the maximum air temperature during heat stress was 2–5 °C higher than that in the control greenhouse (see Supplementary Figure S1). The heat-treated greenhouse experienced 40 days with maximum temperatures exceeding 40 °C, whereas the control greenhouse had 18 such days (see Supplementary Figure S1). The differences of minimum temperatures between heat treated and control conditions were more varied than the maximum temperatures (see Supplementary Figure S1). The minimum temperatures in heat treated greenhouse were 2–10 °C higher than that in the control greenhouse. The diurnal temperature variation for heat-treated and control greenhouses were 18.34 and 15.64, respectively.

Plants were regularly irrigated and fertigated using a drip irrigation system. The nutrient solution A (N 5.5%, K 4.5%, Ca 4.5%, B 0.00014%, Fe 0.05%, Zn 0.0001%, and Mo 0.0002%) and B (N 6%, P 2%, K 4%, Mg 1%, B 0.05%, Mn 0.01%, Zn 0.005%, and Cu 0.0015%) (Mulpure, Daeyu, Seoul, Republic of Korea) were used for the fertigation. The relative humidity in both greenhouses was maintained between 50 and 85%.

Morphological, physiological, and nutrient uptake characteristics were regularly collected through experimental days. The experimental methods have been modified from the previous study^33^. The detailed methods were described in the following sections, including “Collection of morpho-physiological traits and yields of three hot pepper genotypes” and “Calculation nitrogen use efficiency” sections.

### Collection of morpho-physiological traits and yields of three hot pepper genotypes

After subjecting three hot pepper accessions to heat treatment, plant height and stem thickness were measured using a ruler and a digital caliper (CD-20APX, Mitutoyo Co., Ltd., Kanagawa, Japan) on days 0, 2, 4, 11, 25, 34, 41, 56, and 75. The plant height (cm) was measured from the bottom of the stem visible above the soil to the top, and the stem thickness (mm) was measured from the bottom of the stem visible above the soil. Measurements were taken for 3–5 plants per replicate to improve accuracy. The plants were twice on 34th and 75th days after heat treatment for measuring yield components. After harvest, the fresh weights (g) of the fruits, leaves, and stems were documented, along with the determination of the total leaf area index (LAI). Using an LAI integrator (LICOR-300, Lincoln, NE, USA), the LAI of each plant per square meter of crop coverage was determined. Subsequently, the harvested samples were dried at 70 °C, and the dry weights (g) of the stems, leaves, and fruits were measured. The moisture contents of the plants were determined by measuring their fresh and dry weights. Subsequently, the distribution of dry matter was calculated based on the dry weight data for each part of the plants.

### Leaf gas exchange and chlorophyll fluorescence

Using a gas exchange instrument of the model LI-COR LI-6800 (LI-COR, Inc., Lincoln, Nebraska, USA), measurements of the net photosynthetic rate (A, µmol m^−2^ s^−1^) were taken on newly fully expanded leaves at multiple time points: 0, 2, 4, 11, 25, 34, 41, 56, and 75 days following the application of heat treatment. The measurements were carried out between 10:00 and 14:00. The temperatures of the LI-COR chambers were set at 25 °C for the control group and 35 °C for the group subjected to heat treatment. The intensity of light was maintained at 600 µmol m^−2^ s^−1^, and the concentration of CO_2_ was set at 400 µmol m^−2^ s^−1^ CO_2_ with a relative humidity of 60% in the greenhouse conditions. Leaves were exposed to varying degrees of irradiation for 4–5 min until the CO_2_ uptake curve stabilized, and subsequently, data were collected.

Chlorophyll fluorescence data were collected concurrently with net photosynthesis rate measurements. The Photon system Instrument (FluorPen, FP 110, PSI, Drasov, Czech Republic) was employed to assess the photochemical efficiency of PSII (Qy, Fv/Fm) on newly fully expanded leaves following a 15-min period of dark adaptation. Subsequently, saturating light was applied at a rate of 3000 µmol (photon) m^−2^ s^−1^, with actinic light set at 1000 µmol (photon) m^−2^ s^−1^, and measurements were obtained at 3000 µmol (photon) m^−2^ s^−1^.

### Determination of electrolyte leakage potential in seedlings leaves under heat stress

Electrolyte leakage was assessed from fully expanded foliage collected at days 0, 2, 4, 11, 25, 34, 41, 56, and 75 following heat treatment, with three replicates per accession. Leaf discs measuring 5.5 mm in diameter were procured from diverse plants of each accession utilizing a cork bore punch. The punched samples were subsequently deposited into 15 mL tubes containing 10 mL of deionized water and subjected to incubation on a shaker at 25 °C for 30 min. The electrical conductivity (EC1) of the aqueous solution was gauged employing a STARA-HB conductivity meter (Thermo Orion, Waltham, MA, USA). Subsequently, the tubes were exposed to a boiling water bath (100 °C) for 20 min, and measurement of the electrical conductivity (EC2). The ultimate EC content was computed as the percentage ratio of EC1:EC2.

### Calculation nitrogen use efficiency

Plants were harvested on the 34th and 75th days after heat treatment, followed by drying and grinding for nitrogen analysis. The Kjeldahl method was used to determine the total nitrogen content of dried fruits, stems, and leaves. Approximately 1 g of each ground sample was placed into 300 mL glass tubes and then subjected to digestion using a Kjeldahl digestion system (SH420F, Hanon, China) with 15 mL of concentrated H_2_SO_4_. The digested samples were subjected to distillation with a small amount of NaOH using a distillation apparatus (K9840, Haineng Scientific Instrument Co., Ltd., Shandong, China). Following distillation, 0.1 N HCl was gradually added to the samples for total nitrogen content determination. For a more detailed protocol, refer to PanReacAplliChem^34^.

To find the total N (g/kg) can be calculated with the following equation:

$$ \frac{{\left( {ml\,HCl_{sample} - ml\,HCl_{bland} } \right) \times \left[ {HCl_{con} } \right] \times 14.01 \times 100)}}{1000 \times weight\;of\; samples\left( g \right)} $$

Nitrogen use efficiencies (NUE) of fruit (f) and biomass (b) for all varieties grown in both control and heat treatment greenhouses were calculated with the following equation:

$$ NUE_{f} \left( \% \right) = \frac{{{\text{Total}}\;{\text{ N}}\;{\text{ yield}}\;\;{\text{in }}\;{\text{fruit}}}}{{Total \;N\;\;accumulation\; \left( {soil + fertilizer} \right)}} \times 100 $$

$$ NUE_{b} \left( \% \right) = \frac{{{\text{Total}}\;{\text{ N}}\;{\text{ yield}}\;{\text{ in}}\;{\text{ biomass}}\;\left( {{\text{stem}} + {\text{leaves}}} \right)}}{{Total\; N\; accumulation\; \left( {soil + fertilizer} \right)}} \times 100 $$

### Statistical analysis

Split plot ANOVAs were performed to test the effects of heat treatment and accession on each of plant height, net photosynthesis rate, photosynthesis efficiency, and leaf leakage rates, NUEB, and NUEF. The heat treatment and accession were treated as fixed factors. Treatment, days, accession, and interactions between them were tested. Pearson correlation procedures were conducted to analysis the relationships between fresh fruit weight with other morphological characteristics over three accessions in control and heat treatment greenhouses in two different heat stress periods. All statistical analysis were performed using the SAS program (SAS 9.4, NC, USA).

## Results and discussion

### Physiological, morphological, and yield measurements

**Figure 1:**
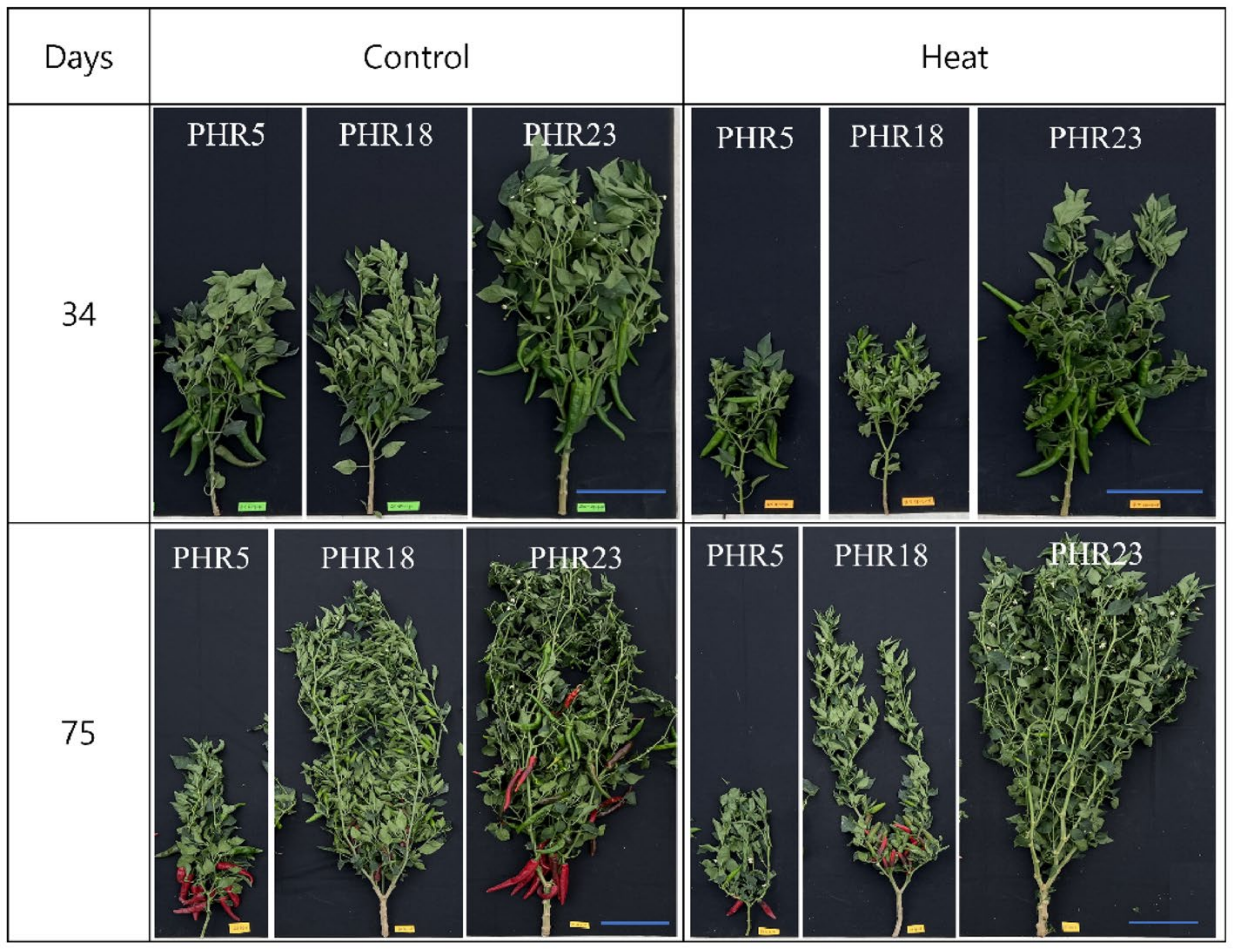
The effects of prolonged heat stress on plant growth of three hot pepper accessions, including PHR5, PHR18, and PHR23 for 34 and 75 days of heat treatments. Blue bars: 20 cm.

Prolonged heat stress effects on the plant growths of three accessions, including PHR5, PHR18, and PHR23, of hot pepper were shown in Fig. 1. As shown in Fig. 1, the morphological and yield measurements of three accessions showed a considerable variation in heat tolerance at two growth periods, including 34 days and 75 days of heat treatments.

**Figure 2:**
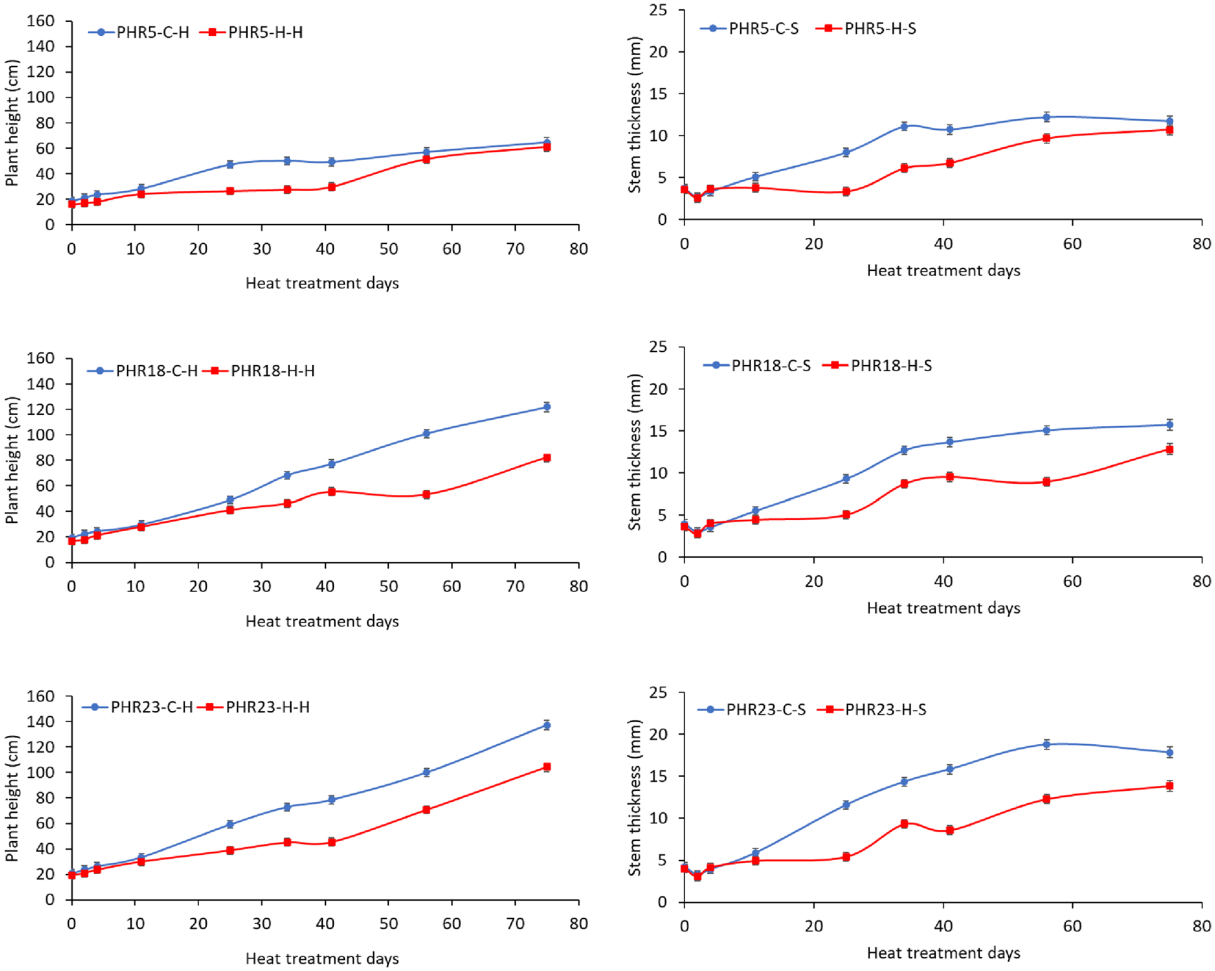
Plant heights and stem thickness of three hot pepper (*Capsicum annuum L.*) accessions, including PHR5, PHR18, and PHR23, treated by heat stress for 75 days. The blue line indicates hot pepper grown under control condition, while the red line indicates the accession grown in heat stress condition. The black bars indicate standard error.

The plant heights and stem thickness of three accessions were continuously measured during the heat treatments for 75 days. In overall, heat stress negatively affected on plant heights and stem thickness of three hot pepper accessions (Fig. 2). Under heat treatment condition, in comparison with control condition, vegetative growth (including plant height and stem thickness) was severely retarded approximately about for 40 days after heat treatment (Fig. 2). After 40th days of heat treatment, the plants tended to regrow (Fig. 2). For example, the accession of PHR23 retarded its growth up to 40th days of treatment, and its growth rate was same as the growth rates of plants in control condition after 40th days of treatment. Like PHR23, other accessions have similar growth patterns under heat stress condition, but their plant heights and stem thickness were smaller than PHR23.

This phenomenon may be related to heat priming that plant store past heat stress information to prepare future heat stress by modulating its biochemistry and physiology by regulating genetic information^35^. The accessions may change its morphological characteristics during the first 40 days after heat treatment to avoid the further possible heat damages. For example, during the initial 40 days of heat treatment, PHR23 decreased its leaf area, height, and stem thickness to have less surface area and leaves in the direct sun, resulting in preventing water loss as well as retaining plant body temperature cooler. Thus, after stress priming, PHR23 may have better tolerance levels in later heat stress exposures.

Morphological variations were observed among three genotypes. PHR5 accession had relatively higher harvest index in range of 0.57–0.77 than other accessions, which means PHR5 produced more fruits than aboveground biomass. In control condition, PHR23 had lower harvest index (around 0.36) in first month, but the accession increased fruit yield in second period, resulting in having the highest harvest index value of 0.66 among the accession. PHR18 had similar growth pattern like PHR23 as the accession produced more biomass in the first period and increased fruit yields in the second period. In control condition, since PHR5 produced more fruits than its biomass, the moisture contents of PHR5 were higher than other accessions. However, in overall, the plant size of PHR5 was the smallest among the accessions, while PHR23 had the greatest plant size (Fig. 2 and Table 1).

**Table 1:** Effects of prolonged heat treatments on fresh weight (g), fresh fruit weight (g), harvest index, leaf area index, and moisture content of three hot pepper accessions, including PHR5, PHR18, and PHR23, in heat treatment and control conditions during two different time periods (34 days and 75 days).

Hot pepper varieties	Heat stress days	Control					Heat stress					Yield difference (%)	
		Total fresh weight (g)/plant	Fresh fruit weight (g)/plant	Harvest index	Leaf area index	Moisture content (%)	Total fresh weight (g)/plant	Fresh fruit weight (g)/plant	Harvest index	Leaf area index	Moisture content (%)	Total Control	Fruit
PHR5	34	385	263	0.68	1.04	86.91	115	88	0.77	0.25	82.31	− 70	− 67
PHR18	34	228	83	0.36	1.00	85.42	125	60	0.48	0.30	87.84	− 45	− 28
PHR23	34	623	225	0.36	3.34	85.23	453	283	0.63	1.14	88.04	− 27	25
PHR5	75	1172	686	0.59	0.81	92.66	259	147	0.57	0.54	85.99	− 78	− 79
PHR18	75	1609	875	0.54	2.60	89.21	835	414	0.50	2.08	85.08	− 48	− 53
PHR23	75	1945	1275	0.66	2.94	85.61	1687	480	0.28	1.94	85.49	− 13	− 62

Yield differences (%) of total weight and fruits were calculated for each accession in each heat-treated period.

Among the three accessions, the PHR23 showed more tolerant to heat stress than other two accessions (Table 1). All statistical analysis were shown in Table 2. According to the statistical analysis, all morphological and yield components were significantly differed by treatment and accessions, except for moisture content, plant height, and stem thickness. There were no interactions between treatment and accession, which means that all accessions had similar responses to heat stress. Heat stress had negatively affected on fresh biomass and fruit weights, harvest index, and leaf area in all three accessions. At both 34th days and 75th days of heat treatment, PHR23 had the highest fresh weights of 623 and 1945 g, respectively. And the total yield losses of PHR23 were only between 13 and 27%. The yield loss in PHR23 was mainly contributed by reduction in leaf area, plant height, and fruit yield. PHR23 had leaf area index of 3.34–2.94 under control condition, while the leaf area index was 1.14–1.94 under heat stress condition. At 34th days, the fresh fruit yield of PHR23 increased by 25%, and its harvest index was increased from 0.36 to 0.63 under heat stress condition. However, under longer heat stress treatment, PHR23 significantly reduced its fruit yield by 62% and had harvest index of 0.28. Under longer heat treatment, PHR18 had better harvest index of 0.50, which produced the second highest fruit yield (417 g) under heat treatment condition. PHR5 was most sensitive to heat stress, and its fresh weight losses were 70% and 78% at 34th and 75th days, respectively. Under heat stress condition, PHR23 could produce three times bigger amounts of fruit yield than PHR5.

**Table 2:** ANOVA of the effects of days (D), heat treatment (T), accession (AC), and their interactions on yield and morphological characteristics.

Variables	Fresh total weight	Fresh fruit weight	Harvest index	Leaf area index	Moisture content	Plant height	Stem thickness
Days (D)	< .0001	< .0001	n.s	0.0364	n.s	< .0001	< .0001
Trt (T)	0.0003	0.0003	n.s	0.0052	n.s	< .0001	< .0001
D*T	0.0199	0.0012	0.0169	n.s	n.s	< .0001	< .0001
Accession (AC)	< .0001	0.0179	0.0272	0.0005	n.s	< .0001	< .0001
D*AC	0.0044	n.s	n.s	n.s	n.s	< .0001	< .0001
T*AC	n.s	n.s	n.s	n.s	n.s	0.0005	0.0014
D*T*AC	n.s	n.s	n.s	n.s	n.s	< .0001	0.0481

n.s. indicates not significant.

Since there were significant interactions between days and treatment were found in fresh weight (*P* = 0.0012, Table 2), correlation analysis between fruit weight and other morphological characteristics were studied for each of treatments and days of heat treatment. According to correlation analysis (Table 3), under control condition, fruit weights were significant positive related with total weight (0.95), stem (0.71) and height (0.77) at 75th days. But the fruit weight was negatively correlated with moisture content (− 0.70). Plant with greater harvest index and low leaf area usually had higher moisture contents (Table1). PHR5 produced the least fruits among the accessions, and this cultivar produced significantly smaller leaves and more fruits over biomass per plant, which results in higher moisture contents than other cultivars. Under heat stress condition, fruit weight was significantly positively correlated with total weight and leaf area only at 34th days. When plants exposed to longer heat stress, there were no significant correlations were found.

**Table 3:** Correlation analysis for showing correlation between fresh fruit weight and other morpho-logical characteristics, including total weight, harvest index, moisture content, leaf area index (LAI), stem thickness, and plant height of all accessions, including PHR5, PHR18, and PHR23, treated with heat for 34 days and 75 days and grown in control conditions.

Treatment	Trt. days	Total weight	Harvest index	MC	LAI	Stem	Height
Control	34	0.69	0.67	0.57	0.28	− 0.42	− 0.13
	75	0.95***	0.61	− 0.70*	0.34	0.71*	0.77*
Heat	34	0.99***	-0.05	0.39	0.98**	0.48	0.62
	75	0.51	0.29	0.22	0.47	0.55	0.59

Numbers in bold indicate significant correlation at alpha = 0.05(*), 0.01(**), < 0.0001(***).

### Effects of prolonged heat stress on leaf damage levels and photoperiodic characteristics

**Figure 3:**
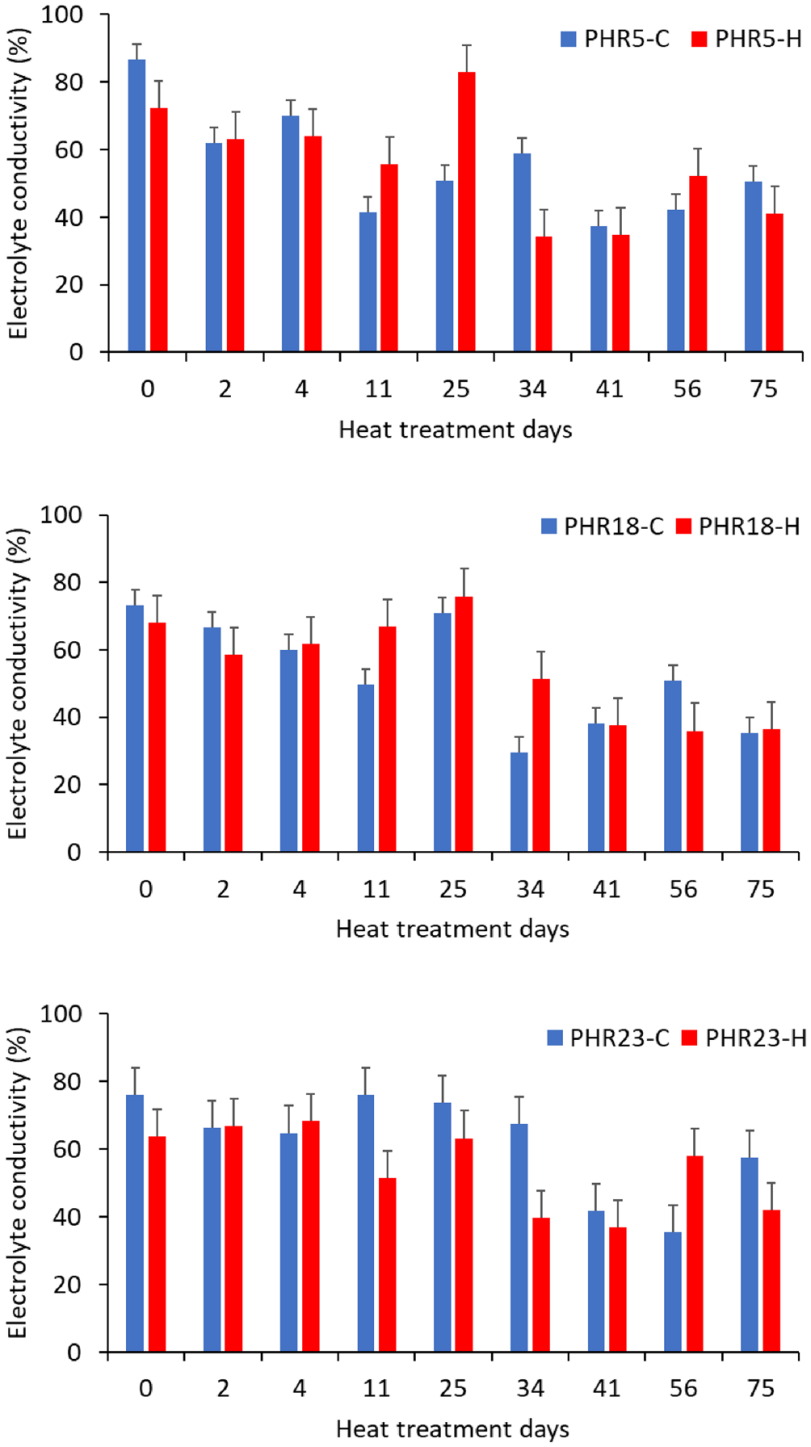
Effects of heat stress on leaf electrolyte leakage rate of three hot pepper accessions, including PHR5, PHR18, and PHR23, for prolonged period in heat stress treatment and control greenhouses. The black bars indicate standard error.

Leaf damage levels were periodically measured electrolyte leakages from leaf discs as an indicator of heat injury after heat treatment (Fig. 3). According to statistical analysis (Table 4), there were significant interaction between heat treatment days and treatment (*P* < 0.0001). The similar patterns of leaf damage level changes across days were obtained for all accessions under heat treatment conditions. In heat treatment condition, the leaf damage levels increased up to 25 days of heat treatment, and lower values of damage levels were obtained from 34th days to 75th days of heat treatment (Fig. 3). Higher heat damages were obtained in control condition from 0 to 2nd days of treatment, while the heat damage levels in heat treatment were mostly higher than control after 4th days of treatment. According to the temperature data measured in greenhouses (Supplementary Figure S1), temperatures in control conditions raised from 0 to 2nd days of heat treatment, while temperatures in heat treated greenhouse were significantly dropped from 0 to 2nd days. This may why higher heat damages in control condition were observed.

**Table 4:** ANOVA of the effects of days (D), heat treatment (T), accession (AC), and their interactions on leaf heat damage levels, PSII efficiency (Qy), net photosynthesis rate (A), and nutrient use efficiency of biomass and fruits.

Variables	EC	A	Qy	NUEB	NUEF
Days (D)	< .0001	< .0001	< .0001	0.0004	0.0001
Trt (T)	n.s	< .0001	0.0347	n.s	0.0351
D*T	0.0048	< .0001	< .0001	n.s	n.s
Accessions (A)	n.s	0.0403	n.s	0.0008	0.0431
D*A	n.s	n.s	n.s	n.s	n.s
T*A	n.s	n.s	n.s	n.s	n.s
D*T*A	n.s	n.s	n.s	n.s	n.s

n.s. indicates not significant.

**Figure 4:**
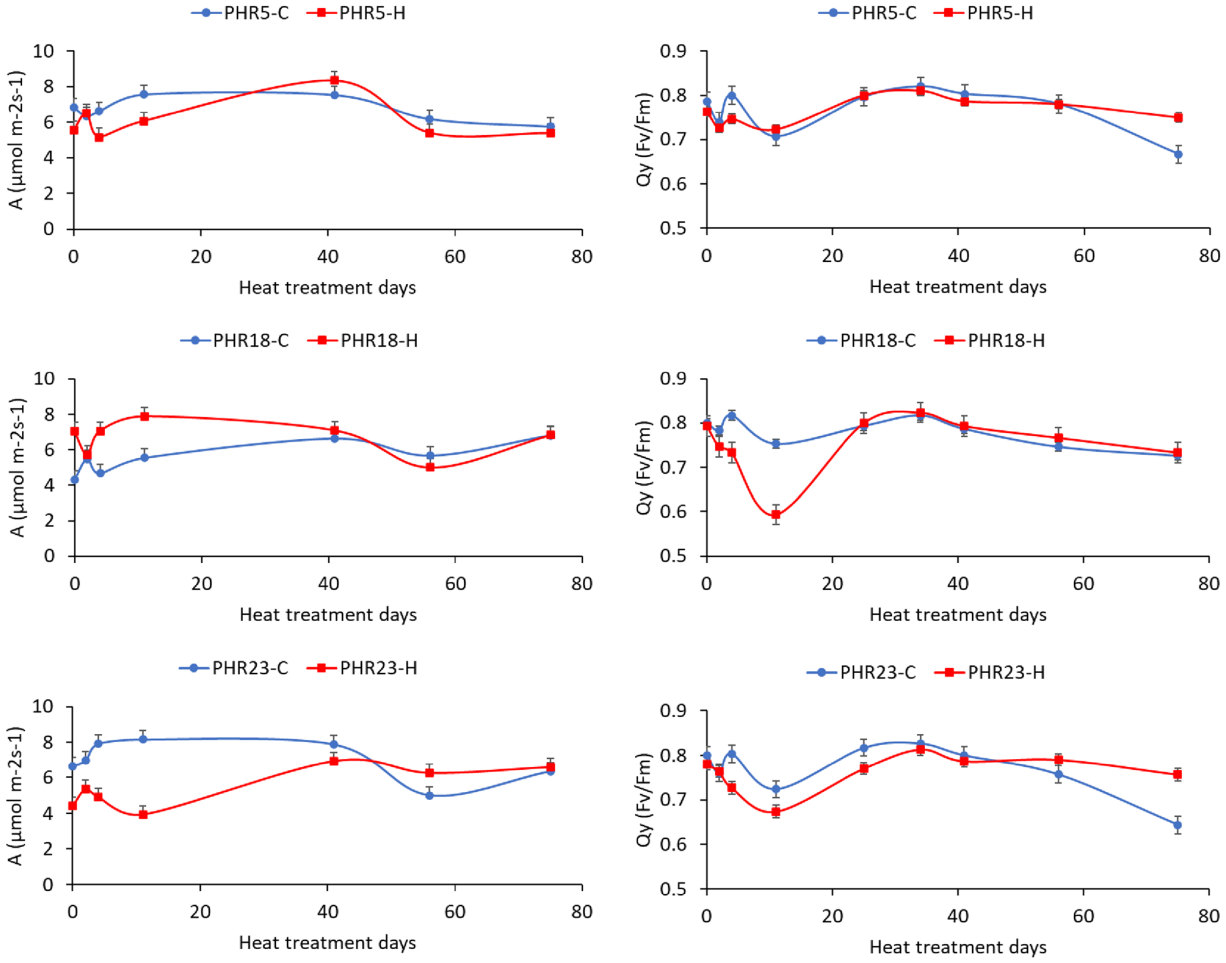
Effects of prolonged heat treatment on the net photosynthesis rate (A) and PSII efficiency (Qy, Fv/Fm) of hot pepper accessions, including PHR5, PHR18, and PHR23, grown in heat-treated and control greenhouses for 75 days. The black bars indicate standard error.

Net photosynthesis rate (A) and photosynthesis efficiency (Qy) were measured periodically for 75 days of heat treatment in both control and heat treatment condition. According to statistical analysis (Table 4), there were significant interactions between days and treatment at *P* = 0.0179 for A and *P* < 0.0001 for Qy. The change patterns for both A and Qy were similar to leaf damage level changes across heat treatment days (Fig. 4). For 30–40 days of heat treatment, the photosynthesis rates under heat treatment days were lower than control condition, and then the photosynthesis rates increased after 10th days of heat treatments and became close to the values in control condition. As photosynthesis rates recovered in few days after heat stress exposures, plant grew back after 40th days of heat treatment (Fig. 2).

### Effects of prolonged heat stress on nitrogen use efficiency

Nitrogen use efficiency (NUE) for biomass (stem + leaves) and fruit parts were estimated using measured nitrogen contents for each body parts (Table 5). According to statistical analysis, there was a significant interaction between days and treatment on nitrogen use efficiency for fruit part (Table 4). Under control condition, the NUE for fruits significantly increased as they grew. At 34th days, the values of NUEF for PHR5, PHR18, and PHR23 were 7.7, 2.8, and 7.4%, respectively, while the values of NUEF at 75th days were 10.4, 34.2. and 52.7%, respectively (Table 5). As shown in "Physiological, morphological, and yield measurements" section, PHR18 and PHR23 produced more fruits in the second heat stress period, which can support the finding that the values of NUEF of PHR18 and PHR23 increased at 75th days. And the harvest index value of PHR5 had not been changed much between 34 and 75th days, thus the values of NUEF of PHR5 at 34th days only increased by 3%. Under heat condition, the values of NUEF were much smaller than NUEF values in control condition. And the values of NUEF at 75th days were only around 5.8–23.8%, while NUEFs in control condition were around 10.4–52.7%. In addition, under control condition, the uptake amounts of nitrogen for biomass decreased as plants used more nitrogen for fruit production in maturity stage (Table 5). For example, NUE ratios of fruit over biomass of PHR18 and PHR 23 were 0.2–0.3 at 34th days, while both NUE ratios were around 0.8 at 75th days. This means that more energy and nutrient were used for fruit productions in maturity stage. When plants exposure to longer heat stress, however, the ratios remained constant between ratios at 34th and 75th days. This referred that plant still used more nutrients to produce biomass production in maturity stage, which can explain how plants increase their biomass and fruit yields after 40th days of heat treatment.

**Table 5:** Nitrogen Use Efficiency (NUE, %) and relative ratios of fruit and biomass NUE of three hot pepper accessions, including PHR5, PHR18, and PHR23, grown in heat-treated and control greenhouses for 34 days and 75 days.

Treatment	Trt. day accessions	NUE (%)				Fruit/biomass	
		34 D		75D			
		Fruit	Biomass	Fruit	Biomass	34D	75D
Control	PHR5	7.7	10.0	18.8	10.4	0.8	1.8
	PHR18	2.8	11.7	28.6	34.2	0.2	0.8
	PHR23	7.4	26.8	42.9	52.7	0.3	0.8
Heat	PHR5	3.3	4.0	5.8	8.9	0.8	0.7
	PHR18	1.7	5.6	16.4	35.6	0.3	0.5
	PHR23	9.4	13.1	23.8	32.8	0.7	0.7

## Conclusion

In this study, the physiological and morphological patterns of three hot pepper accessions treated by prolonged heat stress were analyzed. The three hot pepper accessions were varied with tolerant levels to heat stress. Based on the yield measurements, PHR23 was the most heat tolerant cultivar that produced the greatest biomass and fruit yields among the accession. The total fresh weight of PHR23 in control and heat-treated conditions were 1945 g/plant and 1687 g/plant, respectively. Only 13% of yield has been lost in heat-treated condition. However, PHR5 produced the least biomass among the accessions in both conditions, and PHR5 lost its biomass around 78% in heat-treated condition. PHR18 produced the second largest fruit yields (414 g/plant) at 75th days of heat treatment. But, this cultivar was retarded its growth for a month after exposing to heat stress and significantly grew back around 40th days after heat exposures. The similar growth patterns of PHR18 were also observed in other accessions under heat stress condition. During the first month of heat treatment, all accessions had negative responses to heat stress. However, plant started to regrowth after around 40 days of heat treatment. For example, plant height stopped growing or slowly grew until 40th days of heat treatment, and plant started to regrow after 40th days of heat treatment (Fig. 2). Other morphological characteristic such as stem thickness had similar pattern of plant height. The morphological changes also can be explained by physiological changes (including photosynthesis and cell damage levels). Cell damage levels were significantly reduced after 40th days of treatment, and the photosynthesis rates were significantly increased after 40th days of heat treatments. Plants may modify their cellular parameters from early heat stress exposures to prevent potential heat stress damages, resulting in increasing photosynthesis rates. Under heat stress condition, nutrient uptake amounts for biomass were still high at maturity stage (75th days of treatment), which supported the observed phenomenon that plants started to regrowth after pro-longed heat exposures. However, further studies on molecular mechanisms related to thermos-priming are needed to understand physiological, biochemical, and molecular adjustments in various hot pepper accessions against heat stress. In addition, a pollen viability of pepper is also highly sensitive to heat stress. Thus, in further study, it is necessary to evaluate the pollen viabilities in different heat periods for understanding of the effects of heat stress exposing time on fruit production.

### Supplementary Information

Supplementary Information.

## Data Availability

All data pertaining to the study are either provided within the article or can be found in the supplementary information. Furthermore, the data sets utilized and/or analyzed during the current study can be obtained from the corresponding author upon a reasonable request.
