# Supplementary material for: Comparative heat stress responses of three hot pepper (Capsicum annuum L.) genotypes differing temperature sensitivity
Source: Sci Rep. 2023 Aug 30;13:14203. doi: 10.1038/s41598-023-41418-5 (PMC10468523; doi:10.1038/s41598-023-41418-5)
Supplement: Supplementary file 1 — Supplementary Information. [file 41598_2023_41418_MOESM1_ESM.doc]

Supplementary Information

**Comparative heat stress responses of three hot pepper (*Capsicum annuum* L.) genotypes differing temperature sensitivity**

Min Kyoung Kim^1^, Hyo Bong Jeong^2^, Nari Yu^2^, Bo Mi Park^1^, Won Byoung Chae^1^, Oak Jin Lee^2^, Hye Eun Lee^2^ & Sumin Kim^1^*

^1^Department of Environmental Horticulture and Landscape Architecture, Environmental Horticulture, Dankook University, Cheonan 31116, Republic of Korea; kmklife2@naver.com (M.K.); 32191620@dankook.ac.kr (B.P.); wbchae75@dankook.ac.kr (W.C.), ^2^Vegetable Research Division, National Institute of Horticultural & Herbal Science, RDA, Wanju 55365, Republic of Korea; bong9846@korea.kr (H.J.); ynr7328@korea.kr (Y.N.); ojlee6524@korea.kr (L.O.); helee72@korea.kr (L.H.)

* Corresponding authors

E-mail address: sumin.kim@dankook.ac.kr; Tel: +82-042-550-3644


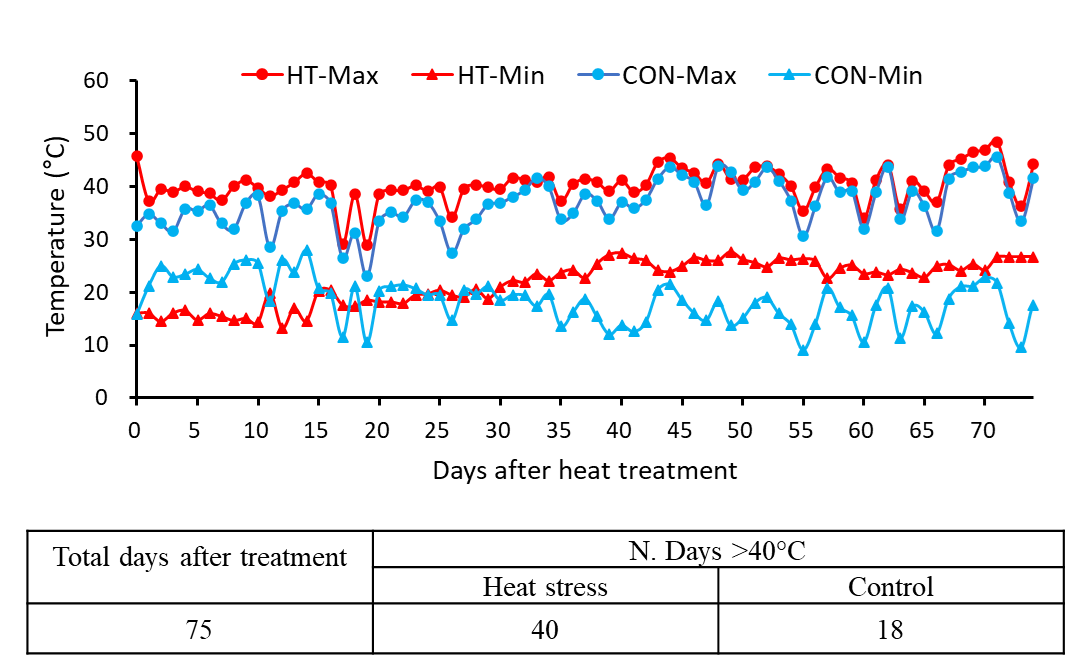


***Supplementary Figure S1.***  *Maximum (Max) and minimum (Min) temperatures across heat treated days in heat-treated (HT) greenhouse and control (CON) greenhouse. Red color indicates the heat-treated greenhouse condition, while blue color indicates the control greenhouse condition. Circle indicates the maximum temperature, while triangle indicates the minimum temperature. Number of total treatment days, and the number of days when the daily maximum temperatures were over 40℃ in both greenhouses during the experiment were presented in the table.*
